# Marking out the clinical expert/clinical leader/clinical scholar: perspectives from nurses in the clinical arena

**DOI:** 10.1186/1472-6955-12-12

**Published:** 2013-04-15

**Authors:** Judy Mannix, Lesley Wilkes, Debra Jackson

**Affiliations:** 1 School of Nursing and Midwifery, University of Western Sydney, Locked Bag 1797, Penrith, NSW 2751, Australia; 2 Family and Community Health Research Group (FaCH), School of Nursing and Midwifery, University of Western Sydney and Conjoint Appointment with Nepean Blue Mountains, Local Health District, Sydney, Australia; 3 Faculty of Health, University of Technology Sydney, PO Box 123, Broadway, NSW 2007, Australia

**Keywords:** Clinical scholarship, Leadership, Nursing

## Abstract

**Background:**

Clinical scholarship has been conceptualised and theorised in the nursing literature for over 30 years but no research has captured nurses’ clinicians’ views on how it differs or is the same as clinical expertise and clinical leadership. The aim of this study was to determine clinical nurses’ understanding of the differences and similarities between the clinical expert, clinical leader and clinical scholar.

**Methods:**

A descriptive interpretative qualitative approach using semi-structured interviews with 18 practising nurses from Australia, Canada and England. The audio-taped interviews were transcribed and the text coded for emerging themes. The themes were sorted into categories of clinical expert, clinical leader and clinical scholarship as described by the participants. These themes were then compared and contrasted and the essential elements that characterise the nursing roles of the clinical expert, clinical leader and clinical scholar were identified.

**Results:**

Clinical experts were seen as linking knowledge to practice with some displaying clinical leadership and scholarship. Clinical leadership is seen as a positional construct with a management emphasis. For the clinical scholar they linked theory and practice and encouraged research and dissemination of knowledge.

**Conclusion:**

There are distinct markers for the roles of clinical expert, clinical leader and clinical scholar. Nurses working in one or more of these roles need to work together to improve patient care. An ‘ideal nurse’ may be a blending of all three constructs. As nursing is a practice discipline its scholarship should be predominantly based on clinical scholarship. Nurses need to be encouraged to go beyond their roles as clinical leaders and experts to use their position to challenge and change through the propagation of knowledge to their community.

## Introduction

In the context of contemporary nursing the crucial need for clinical leadership and clinical expertise (proficiency) abounds. In conjunction with this there is a cry for clinical scholarship. In the late 1990s Sigma Theta Tau International [1] espoused that clinical scholarship was “an approach which enables evidence-based nursing and the development of best practice to meet the needs of clients efficiently and effectively” (p4). This influential international society goes on to state that this clinical scholarship can only flourish if both clinical and academic organisations embrace it. Clinical scholars are viewed as those nurses who are reflective, curious, critical thinkers who contribute to the clinical environment through sharing their knowledge with the broader nursing and general community [1]. In this paper we consider the notion of the clinical scholar, and question if it is any different from a clinical expert or a clinical leader.

## Background

In reviewing the literature on clinical scholarship/scholar no research data-based studies were found. The writings are discussion papers or views of experts in nursing. Mohide and Coker [2] suggest that clinical scholarship in nursing goes beyond expertise, linking it with evidence of practice. As Diers [3] so eloquently states, clinical scholarship is simply “the study of the nature and effect of nursing” (p2). This is affirmed by Roth [4] in a lecture to medical practitioners where he states clinical scholarship refers to clinical practice and includes clinical competence (expertise, proficiency). Thus, as Palmer [5] stated in the 1980s, nurses must bridge the gap between nursing action and nursing thought, or thinking/doing nursing. This reflects the idea of a theory/practice gap: an issue that has long confounded nursing scholars. In the 1990s Walker [6] highlighted the tensions between thinking and doing nursing; and this tension continues, despite increasing numbers of clinical nurses pursuing higher degree education and research [7,8]. These pursuits are often seen internationally as essential criteria for clinical expert positions.

Within the current nursing agenda to improve nursing practice and patient/client outcomes, clinical leadership is often advocated as an essential element [9-11]. Clinical leadership is often linked to solutions for clinical problems that can range from specific clinical problems [12] through to health workplace nursing roles [13]. From an extensive review of research-based literature on the clinical leadership construct, it was found that characteristics of clinical leadership more commonly focussed on practical and technical skills required to lead a team and practise in a clinically competent manner [14]. In this review of research papers it was found that clinical leadership attributes had a clinical focus, a follower/team focus or personal qualities focus; attributes necessary to sustain supportive workplaces and build the capacity and resilience of nursing workforces.

Clinical expertise can be seen as an essential component of clinical leadership. Studies on expertise in nursing became popular after the seminal work of Benner [15] in her description of growth from novice to expert. She described an expert as someone who *knows how* and *knows that* and has both personal and experiential knowledge to use intuition in practice, particularly in decision-making. The nurse expert is someone who has superior performance in their everyday work life [16]. Work into the 1990s and 21^st^ century has focussed on how expert nurses discern cues and make decisions differently to novice practitioners [16-18]. In the context of what role they perform in practise they can be seen as advanced practice nurses. Much research is conducted in this area but Heals [19] sums up their role succinctly as: providing expert clinical practice, facilitating change, disseminating evidence-based practice and improving communication in and beyond the health team. Thornley and West [20] state these nurse experts have an obligation to share their knowledge with novice practitioners and should mentor these nurses.

If scholarship is at the heart of what a profession is, then clinical scholarship must be central to the nursing discipline [21]. The discipline’s philosophy, theory and practice are intertwined and as reflected in Boyer’s ideas, scholarship in nursing can come from four scholarly activities: discovery (research), integration (connections across disciplines), application (practice), and teaching. It is argued by Starck [22] that clinical scholarship, while having significant focus on practice, would also bridge the other three scholarly activities.

Over the decades, scholarship or scholarliness has been discussed and seen as an essential component of the discipline and its practice. Armiger [23] defines scholarliness as being able to argue various points of view, being dedicated and flexible. Others suggest it is being visionary, pursuing excellence, proficiency and mastering systematic knowledge [24,25]. As Kitson [26] states emphatically, “true scholarship is both the breadth and depth of knowledge an individual has in a particular subject area” (p540) and its development is essential to the nursing discipline. Kitson [26] and others [22,24,27,28] emphasise the need for post graduate students in nursing and in particular, doctorates grounded in clinical practice, to build a stronger culture of inquiry and scholarship in the clinical environment. No evidence of research-based data was found in the literature that explores whether nurses in the 21^st^ century see clinical scholarship as essential to their practice or more broadly to their discipline.

In this context, this paper reports the findings from a study in which it was sought to determine clinical nurses’ understandings of clinical scholarship. In this paper the emphasis is on the clinicians’ perceptions of the differences in the construct of the roles of clinical scholar, clinical expert and clinical leader.

## Method

A descriptive interpretative qualitative approach was used in this study which was conducted in three countries – England, Canada and Australia. The methodology utilised was naturalistic inquiry which has the premise that individuals understand the world differently, but by comparing and contrasting these views, a common understanding can be interpreted [29]. The primary focus of the major project was to define clinical scholarship.

### Participants

The participants in the study were nurses currently working in a clinical role and who were students or graduates of the post graduate program in nursing (Master of Nursing or Doctorates). They were recruited through a modified snowballing approach at five universities (one in Canada, three in England and one in Australia).

### Data collection

Data was collected using a semi-structured interview technique with a set of questions relating to how participants would define the characteristics of clinical scholarship and asked if there were any differences between clinical leadership, clinical scholarship and clinical expertise. All interviews were audio taped and transcribed as text.

### Data analysis

The text from the interviews was entered into NVivo [30] and this program was used to manage and sort the text for the participants’ perceptions of the characteristics of the three different clinical roles. The characteristics of the role were compared and contrasted and essential elements of the nursing roles of the clinical expert, clinical leader and clinical scholar were extracted and tabulated for discussion.

### Ethics

This project complies with the Helsinki Declaration [31]. The project was approved by the University of Western Sydney Human Research Ethics Committee, approval No: H8453. All participants volunteered to be interviewed with a written information sheet presented to participants before they signed a written consent form. The project was of low personal risk and to protect their anonymity and confidentiality no personal details are reported in this paper with pseudonyms used to provide a context for exemplars used in findings.

## Results

Table 1 details the characteristics of the participants. The 18 participants for this study worked in three countries: England (13, 72.20%), Australia (4, 22.22%), and Canada (1, 5.56%). As seen in Table 1, there was a spread of ages with an average of 42.2 years with a range of 29 to 67 years. The first qualification in nursing for the group was spread fairly evenly across hospital certificate, diploma or bachelor degree. The most common highest tertiary qualification was a Master in Nursing (10, 55.56%). The average clinical experience of the group was 17 years with a range of 1.5 to 30 years. While two of the participants were working in academic positions, they worked part-time in clinical practice.

**Table 1:** Participant demographic characteristics

**Demographic characteristic**	**n = 18**	**%**
**Gender**		
Male	4	22.22%
Female	14	77.78%
**Age in years**		
Range	29 to 67	
Average	42	
< 30	1	5.56%
31-40	7	38.89%
41- 50	8	44.45%
51 to 60	2	11.12%
**First nursing qualification**		
Hospital Certificate	7	38.89%
Bachelor Degree	5	27.78%
Diploma	6	33.34%
**Highest tertiary qualification**		
Graduate Diploma	2	11.12%
Bachelor Honours	2	11.12%
Masters	10	55.56%
PhD	4	22.22%
**Years of clinical experience**		
Range	1.5 -30	
Average	17	
< 5	3	16.67%
6 -10	2	11.12%
11- 15	1	5.56%
16 - 20	5	27.78%
21 - 25	5	27.78%
26 - 30	2	11.12%
**Clinical area**		
Paediatrics	4	22.22%
Adult critical care	6	33.3.4%
Non-clinical	2	11.12%
Adult general	6	33.34%
**Do you consider yourself a scholar?**		
No	13	72.22%
Yes	5	27.78%

### Marking out the clinical scholar/clinical expert/clinical leader

While the emphasis of this project was defining clinical scholarship, it became apparent that the nurse participants saw distinct differences in being a clinical scholar, clinical expert or clinical leader. These differences are highlighted in Table 2. The ideal for most of the nurses interviewed was for a nurse to be a combination of clinical scholar and clinical leader or clinical scholar with clinical expertise or a combination of all three. The nurse participants felt scholarship was particularly important for a clinical expert. For example: *To be an expert you need to show a certain amount of scholarship* (M3), and, *I think to be an expert you need to be a scholar* (A1).

**Table 2:** Comparisons between clinical expert, clinical leader & clinical scholar

**Markers of role**	**Clinical expert**	**Clinical leader**	**Clinical scholar**
**Parameters of role**	Local	Local	Broad
**Primary emphasis of role**	Practice	Team, communication	Dissemination of evidence for practice – ideal practice
**Knowledge source**	Mostly practice based rather than academic	Experience emphasis with some academic	Academic emphasis but with some clinical experience.
**Knowledge sharing**	Not always shared	Shared locally	Broad sharing
**Practice –research link**	Translate evidence to practice Some do research	Not emphasised	Do practice focused research
**Vision**	Not evident	Yes	Yes
**Motivating others**	Yes	Yes	Yes

There was a sense that while leadership and expertise could be associated with the jobs people hold, there was also recognition that individuals had leadership qualities and were seen as experts, and this was separate from the roles or positional power they had. As one participant indicated:

*there are some people who are clinical experts, and possibly clinical leaders and some of these people I’ve scoped around the UK… who they are and the job they do,* [Which] *will encompass all three roles but others just fit into individual components* (L1).

For some nurses, there was a perception that clinical scholarship and expertise were different but there was some uncertainty about the exact nature of the differences. For example, participant M3 likened the process to a journey:

*… it’s about the journey and when you are an expert, it is about disseminating the knowledge, and clinical scholarship is about gaining the skills on the road to becoming an expert* (M3).

As indicated in Table 2, the nurse participants perceived there were similar and different markers for each of the three roles. The crucial markers were identified as: *parameters of the role, primary emphasis of the role, source and sharing of knowledge, practice-research link, vision,* and *ability to motivate others.* Each of these elements will be discussed.

#### Parameters of the role

It was clear from the interview texts that for the nurse participants the nursing focus of the scholar was, in the main, different from that of the leader or expert. The scholar was seen to have a broad and holistic knowledge of nursing: [the] *Clinical scholar is someone with a breadth of knowledge, engages with nursing and knows how to blend teaching, engaging with the discipline and linking theory and practice* (A4). On the other hand, the nurse participants saw a leader or expert in a very local clinical arena with specific knowledge and skills for that area of practice. They saw an expert as someone with knowledge of a specific field. Another nurse participant thought *clinical leaders often do not have as broad a view of nursing as a scholar* (A4). However, one nurse participant saw a leader’s role usually as having a narrow nursing focus but sometimes could be broader: *clinical leadership is very narrow, it doesn’t necessarily mean the wider clinical community, it may be a very local role, even though it can be broad* (A3).

#### Primary emphasis of roles

For the nurse participants, the three roles had distinctly different emphases. For the clinical expert their primary focus was their specialty practice and providing patient care: *I think an expert is in contact with the clinical practice more than nursing research while the scholar combines nursing research, nursing development and nursing practice* (S1).

The clinical leader was seen as being team focussed while maintaining relationships in and outside the team for effective decision-making and seen as a linchpin in communication for their local area of practice. It is about *getting people on board, they’re managers* (L2); *they are like the nurse unit managers and clinical nurses specialists, they mentor and things like that, but it doesn’t mean they engage in clinical scholarship* (A2). Further, from another participant:

*Clinical leadership is being able to look at the area where you are working, assess it, evaluate it and that’s ongoing and it’s about leading people to better practice, innovation in practice… always helping… it’s going to be about change and collaborating… leading people through processes. It’s about people* (M1).

The clinical scholar is focussed on gaining evidence of nursing practice outcomes with an emphasis on disseminating this knowledge:

*It is having knowledge and the ability in the workplace for it. To disseminate what you know to a very broad audience and to be peer reviewed on what you write or speak and it’s like it cannot come by itself, it has to be in the broader nursing community* (C1).

#### Knowledge source/knowledge sharing

Clinical leaders and experts were seen by the nurse participants as gaining much of their knowledge for their role from practice. Conversely, the clinical scholar was considered to be more academically focused in gaining their knowledge base, while still drawing on the knowledge gained from their own clinical experience. The participants saw an interdependence of their knowledge and its source.

*I think the expert has a lot of experience and therefore knowledge in their clinical field whereas the scholar has a better overview of how things connect… they depend on each other; the expert in terms of experience … the scholar in terms of broader knowledge (M1*).

For another nurse participant, *the expert may have experiential knowledge but they did not always reflect on what they do … an important process, sometimes you do it informally but unless you discuss with others, write it down, that sort of formal stuff is important* (L2). This writing and formal dissemination outside the clinical arena was only noted in the interviews when the nurse participants were discussing the clinical scholar.

Four of the nurse participants often felt the clinical expert was not always willing to share their expert knowledge or skills, preferring to keep it to themselves:

*some experts are very powerful in the profession. You know, knowledge is power, sometimes in our profession I run into people whose main goal is to control knowledge because that makes them very powerful in their workplace and they don’t share. For example, I worked on a specialist floor and there was an extended nurse coordinator for palliative care, pain and symptom management and yet not a single nurse on that floor knew what she knew* (C1).

This idea of holding on to knowledge was in conflict with how many of the nurse participants saw the ideal clinical expert as someone sharing and providing a new generation of nurses with access to their expert clinical knowledge.

Ideally, the clinical leaders and scholars were seen as very willing to share and help others so they could grow. For the leader this sharing was usually within the organisation or their specialty. The clinical scholar would disseminate to the broader nursing and public community through teaching and various forms of media such as writing, speaking and policy development. As one participant relayed, a clinical scholar is able to disseminate *to a very broad audience and to be peer reviewed on what you write or speak and it’s like scholarship cannot take place by you, it has to be in the broader nursing community* (C1).

#### Practice–research link

The nurse participants did not emphasise any practice research link for the clinical leader role. Although they saw them as gaining knowledge from academic sources they did not, in the main, see translation of research into the leadership role. *They wouldn’t be interested in research, they want to do basic clinical work but they would not be interested in looking at advanced knowledge* (M4).

The clinical experts were seen to translate research outcomes into practice and this was an important element of the role. They were also seen as doing research projects but often not disseminating this to the broader community.

*I would expect …[a clinical expert]… to translate knowledge generated by research to clinical care, so I would expect for example, a policy to be up-to-date clinically on new and novel ideas or what practice should be, what safety issues are and, for example, then be able to incorporate them into mandatory training* (L1).

The clinical scholar is seen as someone who is there to help clinicians and the clinical expert translates the knowledge from research: As one participant indicated, *the clinical scholar did research which was practice-based and was disseminated and shared with the broader community* (L1). Another participant viewed a clinical scholar as being *research orientated… being able to understand the evidence from research and then take that into practice and that theory knowledge gap has been huge for a long time* (L2).

#### Vision

Vision was seen as an important element of the role for clinical leaders and clinical scholars but was not as evident in the interview texts for the clinical expert role. However, the concept of vision may be linked to what some nurse participants recounted as an important element of the expert – the risk-taker and allowing others to take risks. For example, one clinical expert *gave us a licence to learn and it was OK that we didn’t learn the way she learnt* (M1). The concept of risk-taker, although not verbalised, was also evident in the nurse participant’s perception of the role of the clinical scholar and leader, with such terms as *foresight, authority to change, create for yourself, set goals for change* (M2). For the clinical leader, *they need to share a vision with people, offer them something they see as being of value, more face-to-face value than maybe the scholar whose vision may be detached from the clinical area* (C1).

#### Motivating others

All roles had an important element of motivation. For example: *management is just following orders and applying a system, applying instructions, applying theories. Leadership is kind of different in some way where you have to inspire; you have to motivate your followers* (S2). One clinical expert was seen *as a breath of fresh air, she was fantastic, inspiring, because what she didn’t know she looked up and she would come back and tell you. She motivated you to know…you would be looking things up to impress her* (A1). As one nurse participant reflected – a clinical scholar herself.

*While they … [clinical scholars] are motivating they also need a workplace that promotes and encourages scholarship, in that it is valued, there is no value on it in places I’ve worked. I now see it is important to develop my team’s scholarly ways. Scholarly academic pursuits have changed me and I want to inspire other clinicians to set goals and make a difference organisationally and clinically* (L1).

## Discussion

In their perceptions of the clinical expert, the participants in this study saw this nurse as similar to that proposed by Thornley [32], that is, as a role model who encourages, inspires and assists others to reach clinical competence. These experts have gone beyond the ‘doing nurse’ described by Walker [6]. They do link thinking and doing to improve nursing practice and patient care. In this way, they also embody attributes of leadership.

In the view of most of the participants, the clinical leader is described mostly in the context of hierarchical, positional leadership [33], such as a nurse manager where the process of organising a unit or ward (i.e., managing, making decisions, team building, communication) is emphasised. The participants have a fairly limited view of clinical leadership and their perceptions are varied, with few common views. However, the personal attributes of clinical leadership clearly emerge. Leadership styles and associated personal attributes such as those found with transformational leadership [34,35] find the leader transforming the values of followers to support the vision of the organisation by fostering an environment where there is trust and where the vision is shared. In the clinical leadership literature, a transformational leadership style is often cited as the preferred style of clinical leadership to achieve positive outcomes for nursing staff in clinical settings [36-38]. There were indications of this style of leadership in the clinical leaders discussed by the participants in this current study when describing the need for leaders to have vision and sharing this vision, as well as inspiring and motivating others. These attributes are similar to those identified by Stanley [39] who found clinical leadership to be based on personal values and beliefs of nursing and care. Similar to the current study Stanley found that “the attributes of the clinical leader were clinical competence, clinical knowledge, approachability, motivation, empowerment, decision-making, effective communication, being a role model and visibility” ([39] p 20). In this current study, visibility was not evident for the clinical leaders in the broader community of nursing, but rather in the local ward or unit.

The nurses’ perceptions of the clinical scholar reflect the theoretical work on the construct evident in the literature since the early 1980s [1,3,5,24-27]. The findings reflect the curiosity of the scholar, the linking of theory and practice by doing practice-based research, the development of an environment to share the results of research with a broader community, and the nature and effect of nursing. Motivation and inspiration are also important aspects of this role.

Stockhausen and Turale [40] found these essential features of Australian nursing scholars as perceived by recognised academic and clinical scholars. One essential feature of the clinical scholar is practice-based research where results are disseminated; knowledge shared and translated into practice both locally and to the broader community. These attributes have been highlighted in literature related to clinical scholarship in other health disciplines, for example occupational therapists [41], medicine [42]. There is an emerging emphasis in some nursing curricula to teach clinical scholarship [27,28]. However, it may be a construct that needs to develop from practice rather than theory. An academic research focus by nurses can enhance this scholarship but the research needs to move from the academy to the clinic. It also needs to move from practice-based research to the academy [43].

Nurses in all three roles or their amalgam need to work together so that patient care is enhanced. There is a movement to basing research in practice and involving nurse clinicians by exploring their values of patient care and the context in which this care is being enacted. Practice development units in the late 20^th^Century tended to explore nursing rather than patient care [44,45]. There are a number of movements globally, including Canada [46] and United Kingdom [44,47] that emphasise translation of research to practice. These earlier translation models were very linear based [26,44,48]. More recently, a movement to establish a more action research approach within a transformational practice development framework in the practice arena has evolved with the development of a change culture addressing value development of the practitioners [45].

This approach has been advocated and taken up by a number of health services in Australia. The *Essentials of Care* program propagated by NSW Health [49] is one such program and this has encouraged research to promote patient care coming from the shared values of clinicians. From personal observation of the authors and feedback from clinicians and the health department at local seminars, significant clinical change is taking place with this approach to enhance patient care [50,51]. However, one of the disadvantages of this approach is that there is tendency for the activity to be limited to one local area such as a ward or unit and not be disseminated to the broader nursing community. The participating clinicians see the program as quality assurance exercises and they do not go through the usual research ethics procedure to enable publication. Clinical scholars need to work collaboratively to challenge and change this thinking.

In order to achieve the goals of the roles of clinical expert and clinical scholar, education programs that emphasise these roles and concepts are needed. Nurses who are educated to a Masters or doctoral level are needed in both the academic and clinical arenas [8,52]. These roles and their characteristics, however, need to be developed with a blend of academic and clinician input. Action based research is one method of achieving this.

### Limitations of the study

This study, like most qualitative research is limited by numbers of participants. However, if the findings are seen within the context of constructivism [29] where each of us see the world differently but our combined perceptions often share similarities and differences which enhance our understanding of phenomena. This study has been worthwhile in exploring roles that are part of the everyday lives of clinical nurses and the nursing faculty in the academic world.

## Conclusion

This research has shown that there are distinct markers for each of the three roles of clinical expert, clinical leader and clinical scholar. However, in the context of the busy and complex world of clinical nursing practice, nurses often see these roles as complementary. They are not hierarchical, but rather, they perform within different parameters with different foci. However, the clinical scholar may be seen as someone who seeks a much broader spectrum of evidence and inquiry than the other two but this in no way deflects from the importance of the clinical expert and clinical leader. A model of the *ideal nurse* may, in a sense, be a combination of the blending of all three constructs.

### Relevance to clinical practice

As nursing is a practice discipline their scholarship should be predominantly based on clinical scholarship. Nurses need to be encouraged to go beyond their roles as clinical leaders and experts to use their position to challenge and change through the propagation of knowledge to their community. Health agendas need to provide educational structures and financial support so clinicians can balance nursing as a significant practice discipline.

## Competing interest

The authors declare that they have no competing interests.

## Authors’ contributions

Study design: JM, LW & DJ. Data collection and Analysis: JM, LW & DJ. Manuscript preparation: JM, LW & DJ. All authors read and approved the final manuscript.

## Pre-publication history

The pre-publication history for this paper can be accessed here:

http://www.biomedcentral.com/1472-6955/12/12/prepub
